# Effects of fire frequency on litter decomposition as mediated by changes to litter chemistry and soil environmental conditions

**DOI:** 10.1371/journal.pone.0186292

**Published:** 2017-10-12

**Authors:** Cari D. Ficken, Justin P. Wright

**Affiliations:** Department of Biology, Duke University; Durham, North Carolina, United States of America; Universite de Sherbrooke, CANADA

## Abstract

Litter quality and soil environmental conditions are well-studied drivers influencing decomposition rates, but the role played by disturbance legacy, such as fire history, in mediating these drivers is not well understood. Fire history may impact decomposition directly, through changes in soil conditions that impact microbial function, or indirectly, through shifts in plant community composition and litter chemistry. Here, we compared early-stage decomposition rates across longleaf pine forest blocks managed with varying fire frequencies (annual burns, triennial burns, fire-suppression). Using a reciprocal transplant design, we examined how litter chemistry and soil characteristics independently and jointly influenced litter decomposition. We found that both litter chemistry and soil environmental conditions influenced decomposition rates, but only the former was affected by historical fire frequency. Litter from annually burned sites had higher nitrogen content than litter from triennially burned and fire suppression sites, but this was correlated with only a modest increase in decomposition rates. Soil environmental conditions had a larger impact on decomposition than litter chemistry. Across the landscape, decomposition differed more along soil moisture gradients than across fire management regimes. These findings suggest that fire frequency has a limited effect on litter decomposition in this ecosystem, and encourage extending current decomposition frameworks into disturbed systems. However, litter from different species lost different masses due to fire, suggesting that fire may impact decomposition through the preferential combustion of some litter types. Overall, our findings also emphasize the important role of spatial variability in soil environmental conditions, which may be tied to fire frequency across large spatial scales, in driving decomposition rates in this system.

## Introduction

The speed and extent of litter decomposition in forested systems exerts an important control on both the provision of nutrients to autotrophs and the size of the soil carbon pool [1, 2]. Historically, climactic variables like precipitation and temperature have been thought to exert the strongest control over decomposition rates at the global scale [3, 4], but there has recently been a push to move away from this climate-centric paradigm and incorporate local, biotic regulatory factors into decomposition frameworks [5, 6]. Within ecosystems, the effect of litter quality on decomposition is modulated by local soil environmental conditions and microclimate [7, 8]. Across biomes, litter decomposition may be predominantly explained by litter quality, itself which may be related to the life history strategies of individual species [6].

In disturbed systems, the relationships between litter traits and local biotic and abiotic conditions are likely altered, since frequent disturbances can alter plant tissue chemistry [9] and soil conditions [10], and filter the plant species pool [11]. How disturbance mediates the effects of litter quality and environmental conditions on decomposition remains poorly studied. In general, fast-growing plants produce high-quality litter (i.e. high in nitrogen and/or phosphorus concentration and low in lignin [12]) that decomposes rapidly, whereas slow-growing plants produce litter with higher concentrations of recalcitrant C compounds that decomposes slowly [6, 13, 14]. Consequently, disturbance can indirectly influence litter decomposition if shifts in community composition are accompanied by shifts in litter quality. For instance, fire suppression promotes woody shrub encroachment at grassland-forest boundaries [15], which reduces primary productivity relative to undisturbed grasslands [16]. Large shifts in plant community composition in response to fire frequency have also been observed in South Africa [17], and in southeastern US longleaf pine forests [18], and since vegetation resprouting after fire has elevated nitrogen concentrations [19], decomposition rates may be elevated following fire if decomposers are nitrogen limited.

In addition to indirect effects through changes in litter quality, disturbance may influence decomposition by directly affecting local soil conditions. One study in tallgrass prairie found that historical fire suppression was correlated with reduced soil N availability and soil moisture, but this had no effect of litter decomposition [20]. Nevertheless, disturbances that alter soil moisture or temperature, which are regularly shown to influence decomposition rates [21–23] may alter the kinetics of enzymes involved in the breakdown of organic matter. Previous work has documented shifts in soil enzyme activity in response to the reintroduction of fire in deciduous forests in Ohio [24], which may impact decomposition of soil organic matter. Fire can also impact litter decomposition if decomposer microbe populations shift [25] or if the local microbial community is best-adapted to decomposing local litter [26]. Finally, post-fire changes to nutrient availability in mineral soil [19, 27] may alleviate nutrient-limitation and increase activity of decomposing microbes. Fires that incompletely burn woody biomass may produce black carbon or biochar [28], which may increase soil microbial biomass and alter microbial and invertebrate composition by influencing soil pH and improving localized soil fertility [29–31].

Together, indirect effects on litter quality and direct effects of fire on soil micro-environmental conditions may influence decomposition rates in nonlinear ways, making it crucial to test how current decomposition frameworks fit into frequently-disturbed systems. Despite the prolific research on decomposition, this function remains difficult to predict because many studies are phenomenological rather than mechanistic [5, 32]. Particularly in frequently-disturbed systems, relationships between litter quality, soil characteristics, and decomposition rates may be modified by disturbances. For example, in tallgrass prairie, soil microbial activity was enhanced following burning, but this effect was dampened as the quality of litter inputs decreased with repeated, annual burns [33]. An empirical framework to understand how micro-environmental characteristics and plant litter traits interact to influence local litter decomposition rates [7] may have implications for understanding nutrient provisioning within and across ecosystems.

Therefore, our goal here was to disentangle the effects of litter quality and local soil environmental conditions on litter decomposition rates in a frequently burned terrestrial system. We conducted a reciprocal litter transplant experiment across sites of varying fire frequencies in a southeastern longleaf pine (*Pinus palustris*) forest. To understand whether the effects of disturbance vary across the landscape, we repeated this set up at two landscape positions that differ in soil moisture. Fire-managed systems provide an ideal template in which to assess the fine- and coarse-controls on decomposition, since the local soil environment and plant community composition vary among fire regimes while climatic conditions are stable [34, 35]. We asked (1) Does litter chemistry of frequently burned communities differ from that of infrequently-burned and fire-suppressed communities? (2) How does litter from sites managed with varying fire frequencies differ in early-stage decomposition? (3) To what extent is litter decomposition driven by (a) direct effects of fire on soil environmental conditions (e.g. soil moisture, soil temperature, plant-soil feedbacks, substrate availability), or (b) indirect effects through changes in plant community composition or litter traits? (4) In frequently-burned systems, does litter mass lost via combustion in prescribed burns vary among fire-management regimes?

## Materials and methods

### Study site

Our study was carried out near Fayetteville, NC, USA in a longleaf pine forest on Fort Bragg Military Reservation (35.1391°N 78.9991°W). This area has deep, sandy and sandy loam soils. They are Grossarenic Kandiudults of the Candor seires and Arenic Hapludults of the Blaney [36]. Mean monthly temperature ranges from 6.0°C in January to 27.5°C in July. Mean annual precipitation is 127.5 cm, with much of the precipitation falling in June through September (12.47, 14.71, 11.53, and 10. 26 cm, respectively). The rolling landscape creates a pattern of microtopographically-driven environmental gradients, from upland open forests to riparian wetlands, over short spatial scales (~10 m). The uplands are well-drained and savanna-like, with an open longleaf pine (*Pinus palustris*) canopy and a shrubby understory. Separating the uplands from the low riparian wetlands lining streambeds, the ecotones have dense, shrubby understory vegetation and are often dominated by Ericaceous species. In this study, we focus on the uplands and the ecotones, which both experience frequent fires, but vary in soil environmental conditions and community composition.

Fort Bragg is divided into burn parcels (hereafter “sites”) with independent burn histories (mean site area is 45 ha). Since the 1980s, prescribed burns have been used as a management tool to maintain the longleaf pine forest, and burns generally occur on three-year rotations to maintain habitat for rare and endangered species [37]. As a part of a larger study that manipulated the burn frequency of some sites [35], we targeted nine sites that had been managed with annual, triennial, or fire suppression burn regimes since 2011 (three replicates per fire regime). In sum, our annually burned sites received fire disturbance four times in the past five years, our triennially burned sites received fire disturbance one to two times over the same timeframe, and our fire suppression sites did not burn. We classify sites based on their fire regimes, hypothesizing that many soil environmental characteristics vary among the examined fire regimes (see below). Permission was granted for this field work by the Endangered Species Branch at Fort Bragg. This experiment was carried out on publically owned land and did not involve endangered or protected species.

### Litterbag construction and data collection

To assess the role of litter quality on decomposition rates, we created bags with litter mixtures based on the dominant overstory and understory species of each fire regime. In this way, we created a two-species litter mixture that captured the dominant community traits present in each fire regime. In 2013, we collected senesced plant litter from the dominant overstory species and fresh leaf matter from the dominant understory species in annually, triennially, and fire-suppressed sites. *P*. *palustris* was the dominant overstory species in all fire regimes and both landscape positions. While fires rarely reach into the canopy, the understory vegetation composition is strongly controlled by fire history [35, 38, 39]. Dominant understory species differed across fire regimes and between landscape positions (upland vs. ecotone). In the uplands, the dominant understory species was *Aristida stricta* for annual and fire-suppressed fire regimes, *Quercus marilandica* for the triennial fire regimes. (Although *A*. *stricta* was the dominant understory species in both annual and fire-suppression sites, trait variation may occur in response to different fire regimes, allowing the same species to vary in litter quality.) In the ecotone, the dominant understory species was *Ilex glabra*, *Arundinaria tecta*, and *Gaylussacia frondsa* for annual, triennial, and fire-suppressed regimes. After drying litter, we constructed litter bags from aluminum mesh (2x2 mm pore size). Bags were filled with 2.5 g (dry mass) of each of *P*. *palustris* and the dominant understory species.

In October 2013, we deployed one set of litterbags per litter type (annually burned, triennially burned, or fire-suppressed litter) at each of the three replicate sites within each fire return interval (annually burned, triennially burned, or fire-suppressed. This litterbag deployment scheme was the same at upland and ecotone landscape positions. Each set of litter bags consisted of four replicate bags, one of which was removed every three months for one year to capture temporal decomposition patterns. Litter bags were installed flush with soil to minimize differences in old litter accumulation across fire regimes. Hereafter, we refer to the fire regime of the site in which the litter bags decomposed as the *destination environment*. We refer to the fire regime of the site from which the litter was collected as the *litter source*. Therefore, we had a total of three replicate litterbag sets per litter source and per destination environment. To maintain ecological relevancy, only litter collected from the same landscape position was deployed at each plot (i.e. litterbags deployed in the uplands consisted of only upland, not ecotone, litter). Upon collection, litter from *P*. *palustris* and the understory dominant were separated, dried, and weighed.

Sites not under fire suppression experienced prescribed burns during the year our litterbags were deployed. In order to present decomposition rates on the same time interval for all sites, regardless of whether or not they experienced a prescribed burn, these burned time points were excluded from estimates of the mass remaining after one year of decomposition. Because prescribed fires occurred during the summer, unburned bags incubated during the winter and early spring (October to April) time points. Consequently, decomposition trends estimated from the unburned bags slightly underestimated litter loss over one year (see S1 Fig in Supporting Information), but there was a strong correlation between the estimated decomposition from the full and partial dataset (r^2^ = 0.79, P<0.001).

If burned litter could not be not separated and identified to species, only total mass was recorded, and these data we excluded from species-level statistical analyses. To understand the how interactions between litter traits and fire regime affects litter breakdown, we also compared the mass of litter lost in prescribed fires across litter sources and between local fire regimes (only annually- and triennially-burned destination environments).

### Litter chemistry

To examine litter quality traits related to decomposability, we measured leaf %N and %C of 340 leaf samples collected from annually burned, triennially burned, and fire-suppressed sites in Ames, Anderson (35). Leaves were oven dried at 40°C until a constant weight, then ground finely, encapsulated in tin capsules, and combusted on a Carlo-Erba Elemental Analyzer coupled to a mass spectrometer at the Duke Environment Isotope Laboratory. We were unable to measure traits of either *P*. *palustris* or *Q*. *marilandica* from annually-burned sites and so excluded them from analyses of specific litter traits. Again, we categorize litterbags based on the source of their litter, and refer to *litter source* to describe the suite of vegetation community traits that vary across fire regimes and landscape positions.

### Soil environmental conditions

To characterize site-level soil environmental characteristics, we measured soil moisture, temperature (at 10 cm depth), and pH across our study plots in June—August 2013, prior to litterbag incubation. Volumetric soil moisture (%SM) was measured by inserting a HydroSense Time Domain Reflectometry probe 10 cm in to soil. Soil pH was measured in a 1:1 soil:dH_2_O water slurry. Within a 3x5 m area surrounding each litterbag incubation plot eight soil moisture measurements, four temperature measurements, and two pH measurements were taken on average twice throughout the season.

To characterize site-level nutrient availability for plants, we installed Plant Root Simulator (PRS^™^)-probes (WesternAg Innovations, Saskatoon, Saskatchewan, Canada) in each of our plots in 2012. Each plot had four replicate anion and four replicate cation probes installed. The PRS^™^-probes consisted of a plastic probe with a pre-treated ion exchange membrane that is inserted 15 cm into the soil. The probes continuously adsorb ions throughout the duration of incubation. All PRS^™^-probes were incubated *in situ* for approximately 1 month (26–36 days) prior to litterbag incubations. Upon re-collection, we immediately returned PRS^™^-probes to the laboratory, rinsed them with DI water, and shipped them to Western Ag where they were eluted with 0.5N HCl solution for 1 hour. Eluate was analyzed colorimetrically for NO_3_^−^-N via cadmium reduction and NH_4_^+^-N via nitroprusside reaction on a flow injection analyzer (FIAlab 2600; FIAlab Instruments, Inc.; Bellevue, WA, USA), and for other ions (Al^3+^, Ca^2+^, Fe^3+^, K^+^, Mg^2+^, Mn^2+^, H_2_PO_4_^−^-P, and SO_4_^−^-S) with an Inductively Coupled Plasma (Optima 8300 ICP-OES; PerkinElmer Inc.; USA). We report concentrations of NO_3_^−^-N, NH_4_^+^-N, and H_2_PO_4_^−^-P individually, and sum the remaining cations and anions. Again, we classify incubation sites according to their fire regime and refer to the *destination environment* as a proxy for all the measured and unmeasured environmental conditions that vary across fire histories.

### Statistical analyses

For soil environmental characteristics and plant leaf chemistry, we compared differences between fire regimes and landscape positions with linear mixed effects models using the nlme package (version 3.1) in R [40] and included site as a random effect. We report the degrees of freedom and the t- and p-values of the fixed effects as well as the marginal (fixed effects only) and conditional (fixed effects and random site-effects) R^2^ values of best models, as estimated with the sem.model.fits function in the piecewiseSEM package (version 1.2) in R.

To compare the direct (destination environment) and indirect (litter source) effects of fire on decomposition, we first fit exponential decay curves to unburned time points within each time series using the nlme package [41] in R. By extrapolating decomposition rates from the full time series, rather than using the measured final mass of individual bags decomposing for the full year, we minimized stochastic differences between bags (e.g. slight differences in initial litter mass or packing density) that may have influenced the decomposition of individual bags. We then fit linear mixed effect models to the estimated proportion of litter mass remaining. Upland and ecotone landscape positions differed significantly in decomposition of all litter types, so we analyzed upland and ecotone landscape positions separately. Best models were chosen with AIC. If litter source was a significant predictor, we computed additional regressions with specific litter chemistry traits (%N and C:N) as predictors.

To examine the potential feedback between fire frequency, litter traits, and litter flammability, we compared the mass of litter lost to fire (for sites that burned, i.e. all except fire-suppression sites) among destination environments and litter sources. While we analyzed landscape positions separately for litter decomposition (see above), we grouped them together for analyses of litter combustion because the mass lost to burning did not differ between upland and ecotone landscape positions. To estimate the mass of litter lost to fire (*c*), we fit modified exponential decay curves over the empirical full time series (i.e. four bag collection time points over one year) that included a step loss parameter to account for the mass of litter lost in prescribed burns: *M* = *ae*^*kt*^ − *Bc* where *M* is the mass remaining after one year of decomposition, *a* is the initial litter mass, *k* is the decomposition rate, *t* is time, *B* is a dummy variable (0 or 1) to account for whether or not the site burned during litterbag deployment, and *c* is the estimated mass combusted. As for decomposition in the absence of fire (previous paragraph), we extrapolated decomposition rates from curves fit to empirical data, rather than using the empirical final bag mass, to minimize stochastic differences between bags. We compared *c* between fire regimes and litter sources with mixed effects models as above. Best models were chosen with AIC.

## Results

### Soil environmental conditions and leaf chemistry

Soil environmental characteristics were relatively consistent among fire regimes, and varied considerably between landscape positions (Table 1). Overall, soils were slightly acidic, more so in the ecotone than in the upland (df = 23, t = 4.68, p<0.001), and relatively dry (<10% soil moisture in the uplands, and 10–15% in the ecotone; Table 1). Soils were significantly wetter in the ecotone than in the uplands (df = 134, t = -6.35, p<0.001) and soil moisture differed substantially between sites (i.e. random site effects; marginal R^2^ = 0.13, conditional R^2^ = 0.56), however there was no difference in soil moisture between fire regimes. Soils were significantly warmer in annually burned compared with fire-suppressed sites (df = 6, t = 2.73, p = 0.034) and in the uplands (df = 134, t = 4.83, p<0.001).

**Table 1:** Soil properties of study sites.

		Moisture (%)	Temp. (°C)	pH
*Upland*	Annual	4.82 (0.40)^a^	24.71 (0.46)^a^	6.23 (0.08)^a^
	Triennial	7.09 (0.79)^a^	23.74 (0.33)^ab^	6.17 (0.13)^a^
	Suppression	6.38 (0.67)^a^	22.26 (0.31)^b^	6.07 (0.06)^a^
*Ecotone*	Annual	10.25 (0.79)^a^	23.09 (0.24)^a^	5.52 (0.15)^a^
	Triennial	16.21 (3.58)^a^	22.92 (0.38)^a^	5.52 (0.24)^a^
	Suppression	11.91 (1.44)^a^	21.02 (0.10)^b^	5.83 (0.19)^a^

Mean values are reported (± standard error, SE). Superscripts (a, b, ab) indicate significant differences in environmental conditions between fire regimes within each landscape position.

**Fig 1:**
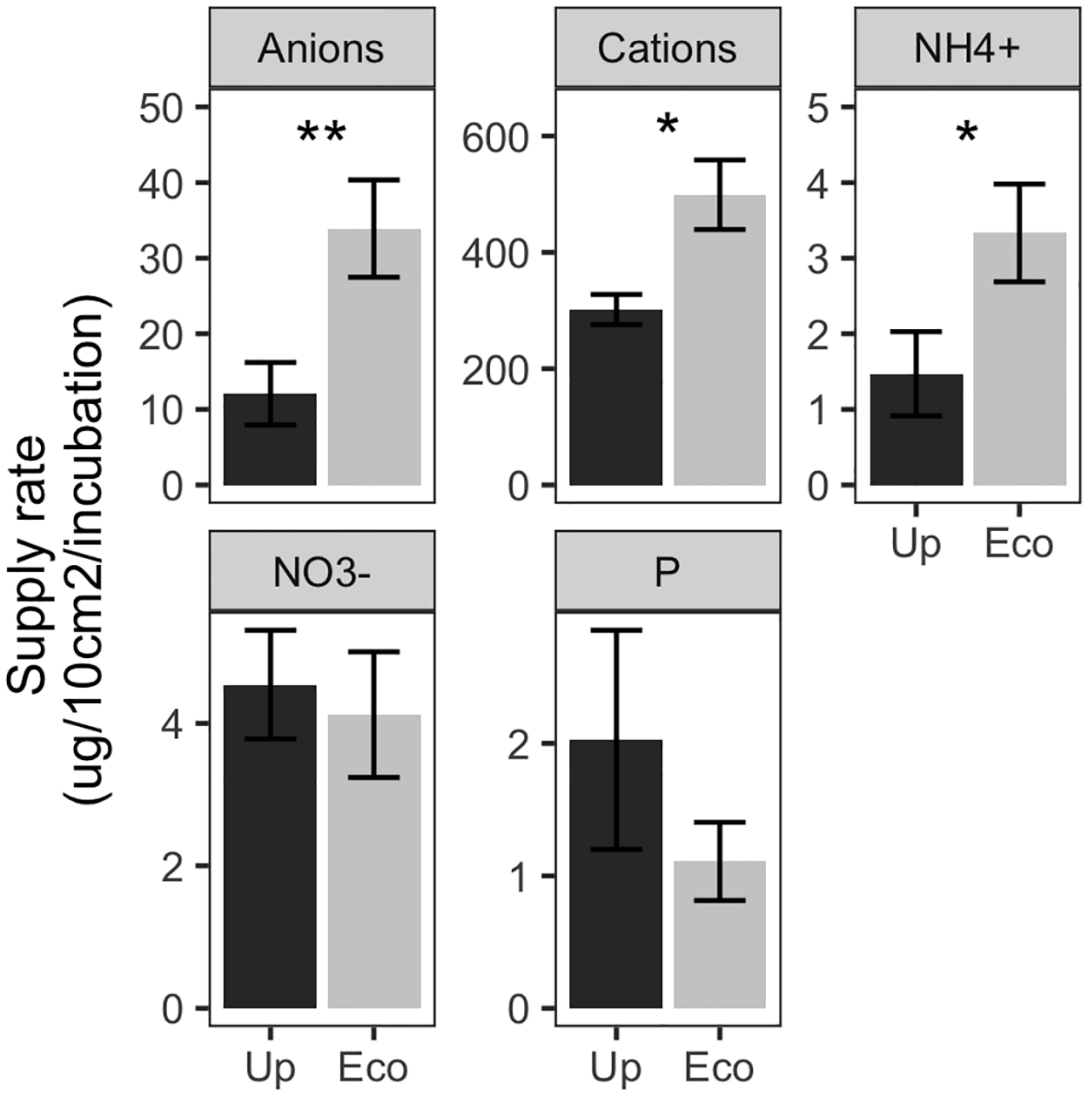
Soil nutrient availability. Nutrient availability (mean ± SE) in upland (“Up) and ecotone (“Eco”) landscape positions. Significant pairwise differences indicated by *** (P<0.001), ** (P<0.01), or * (P<0.05). See text for details on statistical results.

There were no significant differences in nutrient availability across fire regimes. However, a number of ions differed between upland and ecotone landscape positions (Fig 1). Ecotones had higher nutrient availability overall: they had significantly higher supplies of anions (df = 8, t = 4.57, p = 0.002), cations (df = 8, t = 2.97, p = 0.018), and NH_4_^+^ (df = 8, t = 3.08, p = 0.015), and landscape positions did not differ in NO_3_^-^ or PO_4_^3-^ supplies.

Litter carbon and nitrogen content varied considerably between species and across fire regimes. Leaf %N differed significantly across species (ANOVA F_304_ = 277.58; p < 0.001) and burn regimes (ANOVA F_20_ = 4.57; p = 0.023; Table 2). Tukey HSD pairwise comparisons indicated that %N differed between all species (P<0.001 for all except *A*. *stricta–P*. *palustris* where p = 0.018 and *G*. *frondosa*–*Q*. *marilandica* where p = 0.019). *A*. *tecta* had the highest leaf %N across all fire regimes, while *A*. *stricta* and *P*. *palustris* had the lowest (Table 2). Plants from annually-burned sites had higher leaf %N than plants from triennially-burned (df = 20, t = 3.53, p = 0.002) and fire-suppressed sites (df = 20, t = 3.17, p = 0.005). Many individual species showed variation in leaf %N; leaves from annually-burned sites had higher %N than those from fire suppression sites for *A*. *strica*, *A*. *tecta*, and *G*. *frondosa* (Table 2). In contrast, *Q*. *marilandica* leaves had higher %N in suppression than annually burned sites (Table 2).

**Table 2:** Leaf chemistry of target species collected across fire regimes.

	Source	%N	CN
*A*. *stricta*	Annual	1.00 (0.06)^a^*	47.91 (2.81)^a^
	Triennial	0.57 (0.09)^b^*	106.25 (9.88)^b^
	Suppression	0.41 (0.03)^b^	119.20 (8.03)^b^
*A*. *tecta*	Annual	2.77 (0.16)^a^	16.16 (0.86)^a^*
	Triennial	2.06 (0.06)^b^	22.23 (0.74)^b^
	Suppression	1.96 (0.05)^b^	22.96 (0.62)^b^*
*G*. *frondsa*	Annual	1.56 (0.33)^a^*	41.11 (8.47)^a^
	Triennial	1.18 (0.02)^ab^	44.49 (0.88)^a^
	Suppression	1.16 (0.02)^b^*	45.66 (0.96)^a^
*I*. *glabra*	Annual	1.01 (0.06)^a,b^	53.21 (3.08)^a^
	Triennial	0.98 (0.02)^a^*	54.25 (1.24)^a^
	Suppression	1.10 (0.03)^b^*	48.74 (1.30)^a^
*P*. *palustris*	Triennial	0.57 (0.06)^a^	88.68 (9.57)^a^
	Suppression	0.82 (0.03)^a^	58.44 (1.93)^a^
*Q*. *marilandica*	Triennial	1.14 (0.02)^a^	43.15 (0.95)^a^
	Suppression	1.58 (0.09)^b^	30.84 (1.64)^b^

Mean values are reported (±SE). Significant differences among samples sourced from different fire regimes are indicated by different letters (a, b, ab); asterisk (*) indicate marginally (p<0.10) significant differences.

Leaf C:N also differed significantly between species (ANOVA F_304_ = 233.43; P<0.001), and only for some species did leaf C:N differ significantly between burn regime (Table 2). Leaf C:N of *A*. *tecta* and *G*. *frondosa* were relatively consistent and low (Table 2); *A*. *stricta*, on the other hand, exhibited the highest and most variable leaf C:N, ranging from ~48 to 120 (Table 2).

### Decomposition trends across treatments

**Fig 2:**
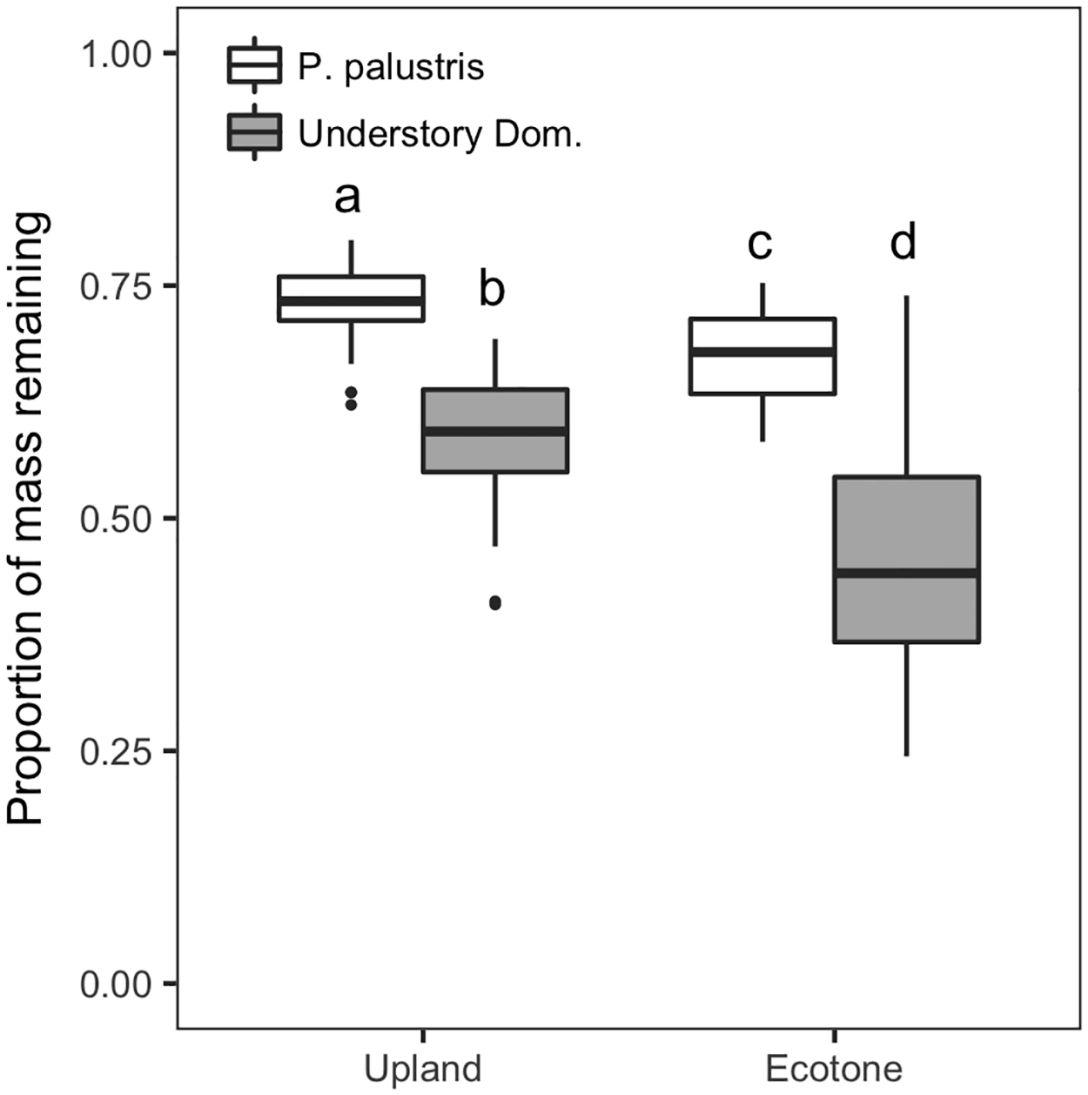
Litter decomposition across landscape positions. Decomposition of both litter types (*Pinus palustris* and dominant understory species) in the absence of fires differed between landscape positions. Significant pairwise differences indicated by different letters.

Across all destination environments and litter sources, 39% (±1% standard error, SE) of litter mass was lost after one year of decomposition in the absence of fires (see S1 Table for decomposition rates, *k*, under each treatment). Decomposition did not differ across fire regimes in either the uplands or the ecotones, but decomposition was faster in the ecotones than the uplands (df = 43, t = 5.01, P<0.001; Fig 2). In the uplands and ecotones respectively, 27% (±<1%) and 33% (±<1%) of *P*. *palustris* litter, and 42% (±<1%) and 55% (±<1%) of understory dominant litter mass was lost after one year of decomposition (Fig 2). The dominant understory species decomposed faster than did *P*. *palustris* in both the uplands (df = 42, t = -9.21, p<0.001; Fig 2) and the ecotones (df = 44, t = -9.21, p<0.001; Fig 2).

Decomposition of the aggregated litter mixture varied both across litter sources and destination environments in both the uplands and in the ecotones. In the uplands, the aggregated litter mixture decomposed significantly faster in triennially burned sites than in suppression sites (df = 6, t = 3.62, p = 0.011), and marginally faster than in annually burned sites (df = 6, t = 2.00, p = 0.092); decomposition of the aggregated litter mixture did not differ significantly among litter sources, although the best model included litter source as a predictor. In the ecotones, decomposition of the aggregated litter mixture differed significantly among litter sources, but not destination environment: litter from triennially burned sites decomposed faster than both litter from annually burned (df = 15, t = 2.42, p = 0.029) and fire suppressed sites (df = 15, t = 3.78, p = 0.002).

#### Decomposition in the uplands

**Fig 3:**
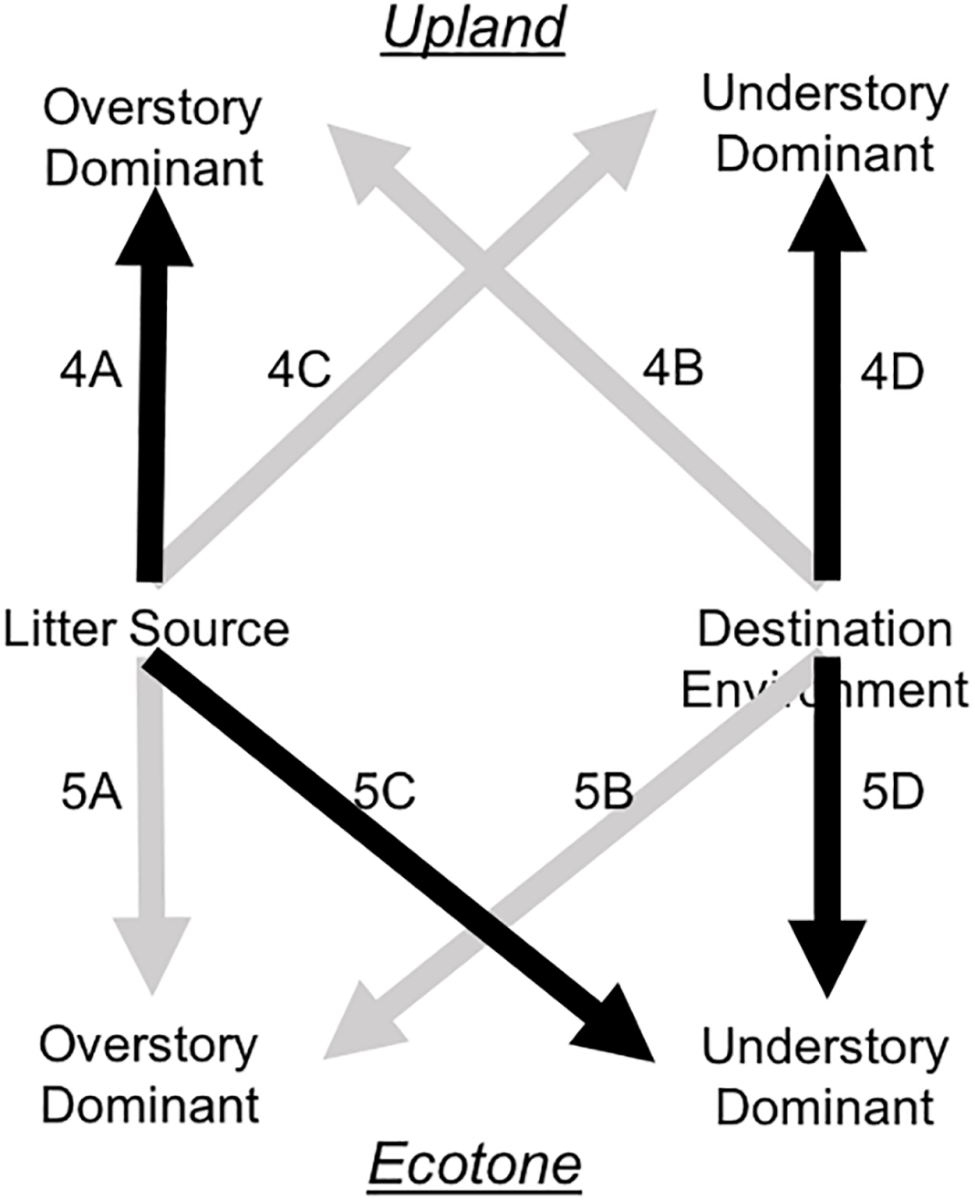
Schematic of effects of fire on litter decomposition. Frequent fire disturbances may affect overstory and understory litter decomposition in upland and ecotone landscape positions indirectly (Litter Source) and directly (Destination Environment). Dark arrows indicate significant effects and light arrows indicate non-significant effects. Codes next to arrows refer to the Fig and panel number displaying the result.

**Fig 4:**
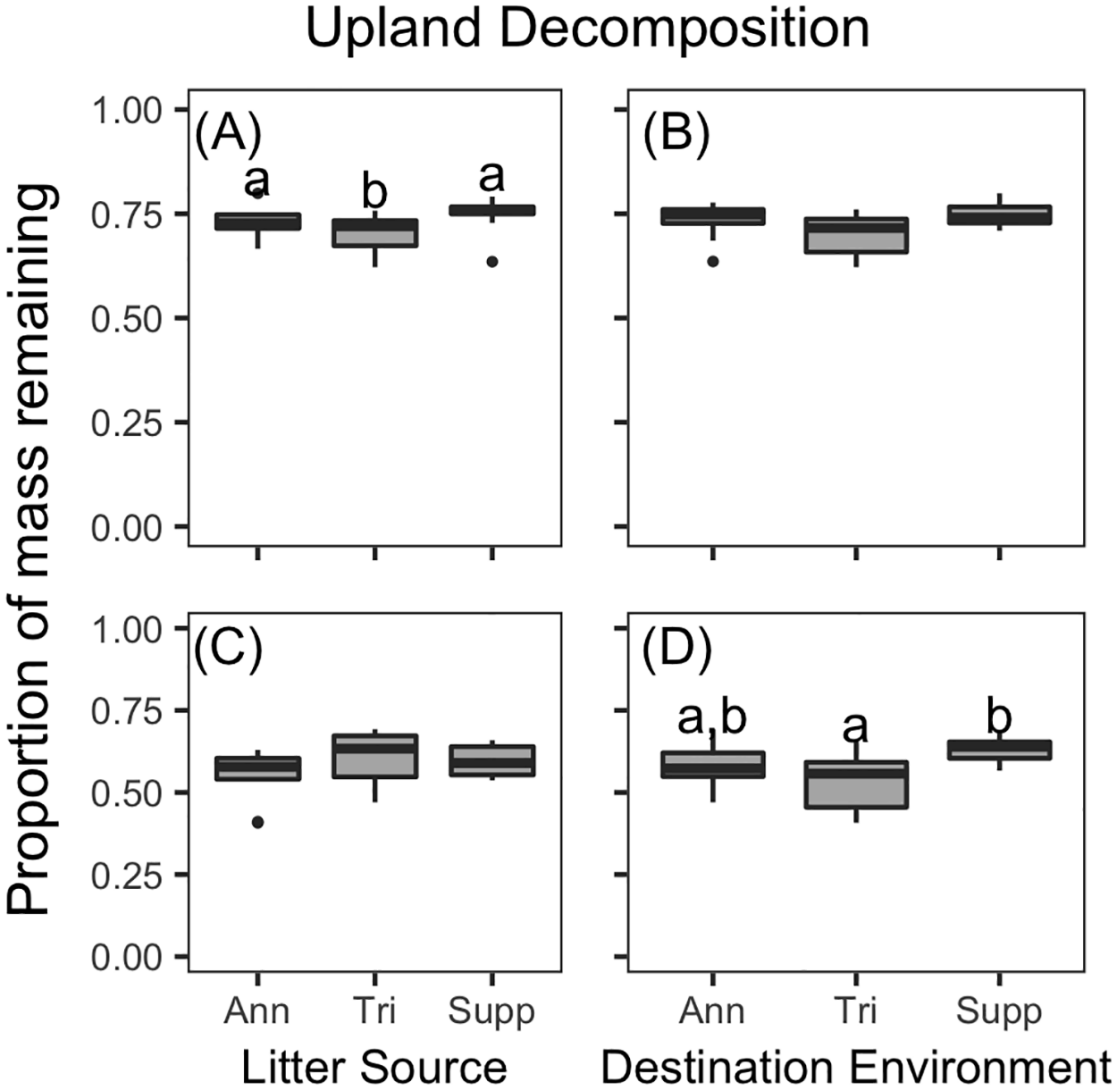
Upland litter decomposition across treatments. Boxplots of mass remaining of litter from the dominant upland overstory, *P*. *palustris*, (A and B) or understory species (C and D) as a function of Litter Source (A and C) or Destination Environment (B and D). Ann, Tri, and Supp refer to annually-burned, triennially-burned, or fire-suppressed litter (or sites), respectively. See Methods for details on which understory species was dominant at each fire regime. Significant pairwise differences indicated by different letters.

Decomposition trends of the individual litter types did not necessarily correspond to those of the aggregated litter mixtures (Fig 3). In the uplands, *P*. *palustris* decomposed significantly slower than all other species (p<0.001 for all pairwise comparisons), and there were substantial differences in decomposition between sites (marginal R^2^ = 0.19, conditional R^2^ = 0.72). Models that included litter sources as categories outperformed those with litter chemistry. Decomposition of *P*. *palustris* litter in the uplands did not differ among destination environments (Fig 4B), but differed significantly among litter sources (Fig 4A): *P*. *palustris* litter sourced from triennially-burned sites decomposed faster than *P*. *palustris* sourced from annually-burned (df = 15, t = 2.42, p = 0.029) or fire-suppressed sites (df = 15, t = 2.42, p = 0.002) in the uplands. Decomposition of understory litter in the uplands differed among destination environments (Fig 4C) but not litter sources (Fig 4D). Understory litter decomposed faster in sites with triennial burn regimes than sites under fire suppression (df = 6, t = 2.99, p = 0.024). Understory litter decomposed similarly across sites (marginal R^2^ = 0.29, conditional R^2^ = 0.29)

#### Decomposition in the ecotones

**Fig 5:**
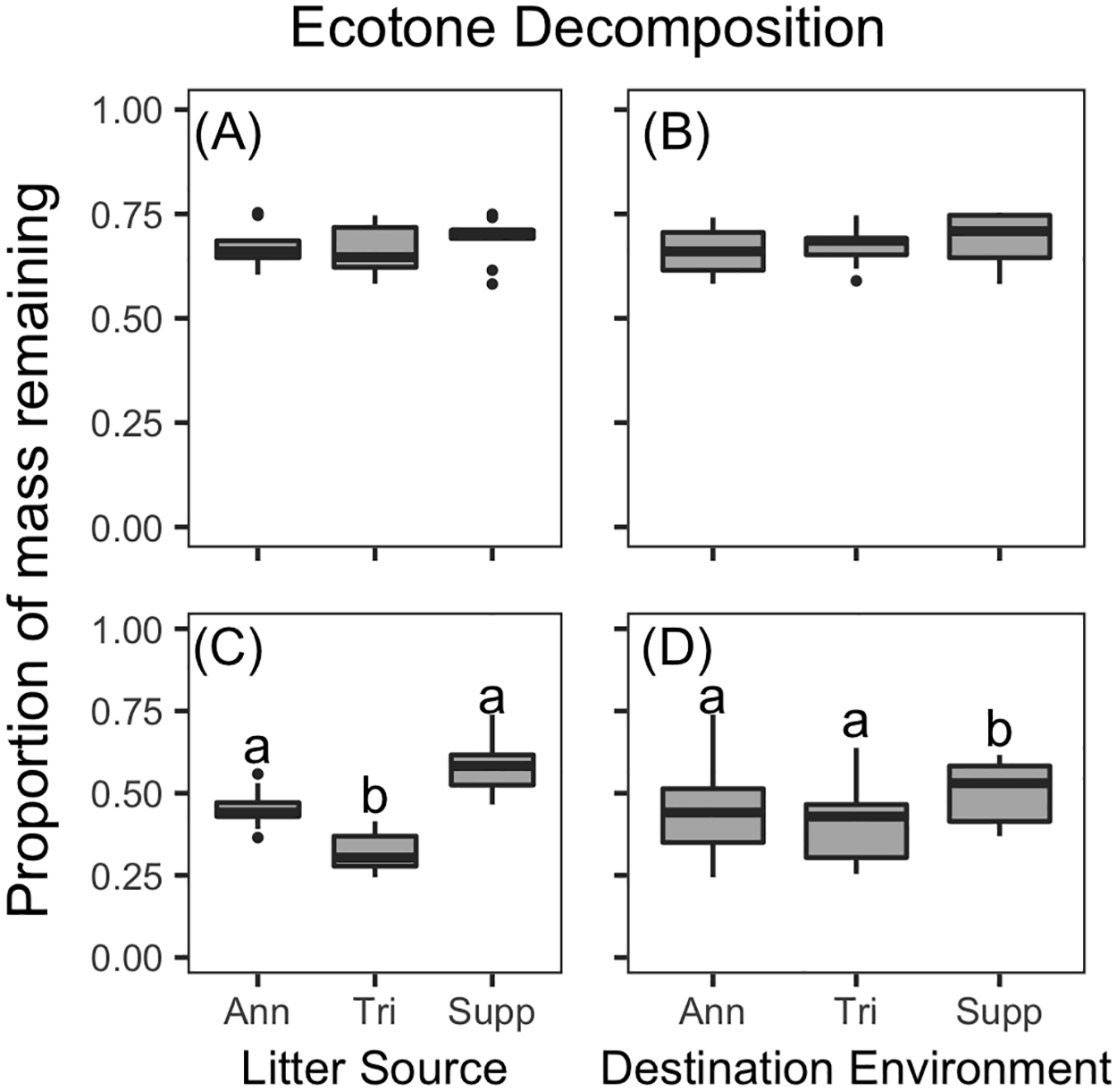
Ecotone litter decomposition across treatments. Boxplots of mass remaining of litter from the dominant ecotone overstory, *P*. *palustris*, (A and B) or understory species (C and D) as a function of Litter Source (A and C) or Destination Environment (B and D). Ann, Tri, and Supp refer to annually-burned, triennially-burned, or fire-suppressed litter (or sites), respectively. See Methods for details on which understory species was dominant at each fire regime. Significant pairwise differences indicated by different letters.

In the ecotones, similar to the uplands, *P*. *palustris* decomposed slower than understory dominant species (p<0.001 for all pairwise comparisons). Overall variability in litter decomposition was higher in the ecotones than in the uplands. In contrast to the uplands, in the ecotones there was no difference in the decomposition of *P*. *palustris* litter from different litter sources (Figs 3 and 5A) or when it decomposed in different destination environments (Figs 3 and 5B). Best models of *P*. *palustris* litter in the ecotone included only the random site effects (marginal R^2^ = 0, conditional R^2^ = 0.55). There were significant differences in the decomposition of understory dominant species in the ecotone. Decomposition of the understory litter in the ecotone differed both among litter sources (Fig 5C) and destination environments (Fig 5D). In the ecotones, understory litter decomposed faster in triennially burned sites than fire-suppression sites (df = 6, t = 2.77, p = 0.033; Fig 5D). Understory litter sourced from triennially burned sites decomposed significantly faster than litter sourced from annually burned (df = 16, t = 5.77, p<0.001) and fire suppression sites (df = 16, t = 11.27, p<0.001; Fig 5C). Models with categorical litter sources outperformed those with litter chemistry traits.

### Fire-driven loss of litter sourced from different fire regimes

**Fig 6:**
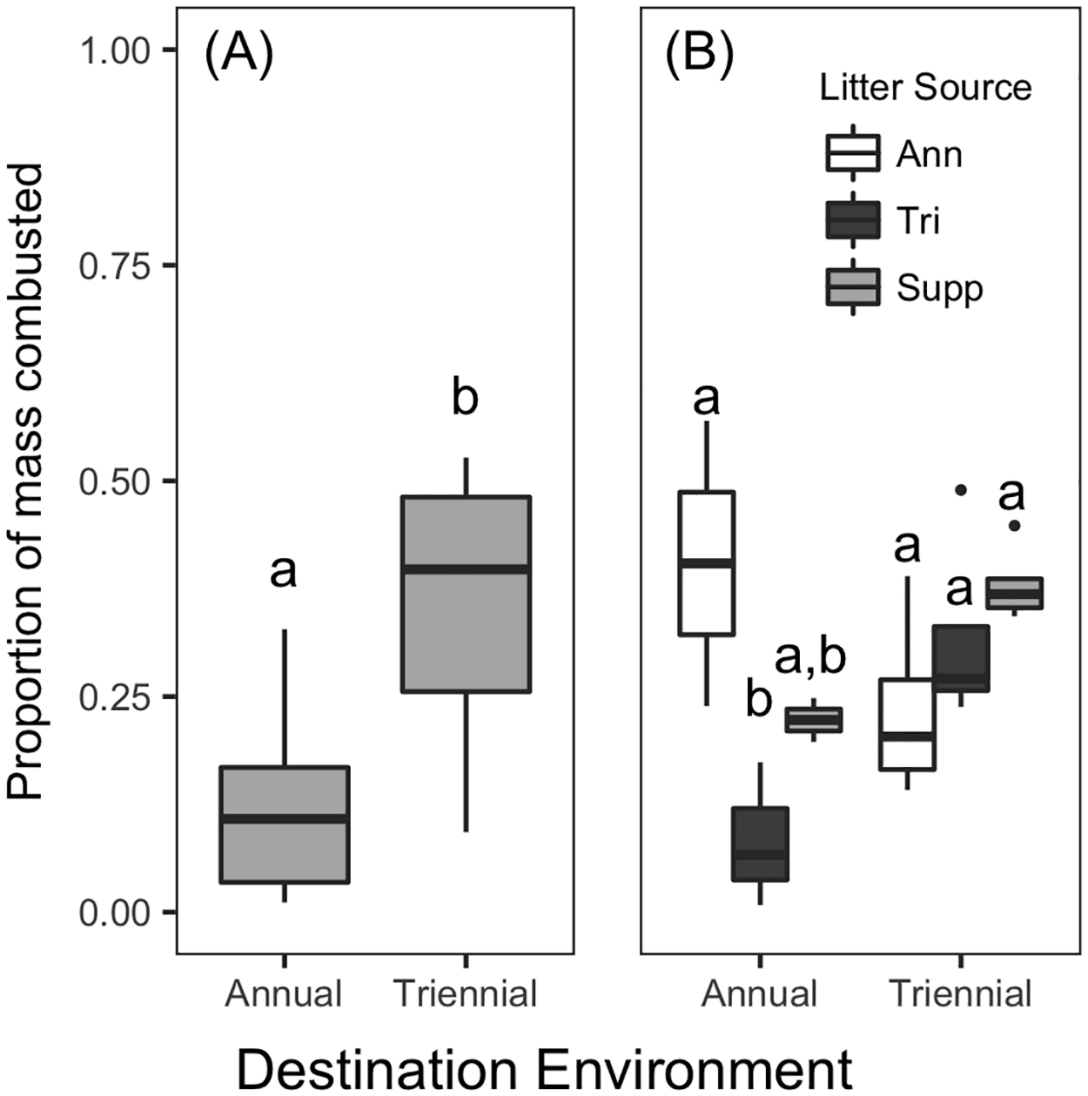
Litter losses during prescribed fires. Proportion of litter from (A) *P*. *palustris* and (B) understory dominant species combusted during prescribed fires. Significant pairwise differences indicated by different letters. In B, comparisons refer only to pairs within a fire regime of the destination environment; refer to the text for statistical details across destination environment fire regimes. Ann, Tri, and Supp refer to litter from annually-burned, triennially-burned, or fire-suppressed sites, respectively.

To examine the direct effects of fire on litter loss, we compared the mass of litter lost in prescribed burns across fire regimes. While decomposition varied between landscape positions, there was no difference in the mass of any litter type combusted in fires between the uplands and ecotones (df = 18, t = 0.95, p = 0.356). For this reason, we grouped litter from both landscape positions in analyses of litter combustion. The fire-driven litter loss of *P*. *palustris* litter was best explained by models that included only destination environment: a greater proportion of *P*. *palustris* litter was combusted in triennially-burned sites compared to annually-burned sites (df = 4, t = 4.38, p = 0.012; Fig 6A). For understory litter, the destination environment, litter source, and their interaction were all included in the best model estimating the proportion of litter combusted in prescribed fires. A greater proportion of annually-burned litter combusted in fires as compared with triennially-burned litter (df = 13, t = 3.50, p = 0.004), and a marginally greater proportion of annually burned litter combusted in fires as compared with fire-suppressed litter (df = 13, t = -1.81, p = 0.094; Fig 6B). There was no significant direct effect of the destination environment, but there was a significant interaction between the destination environment and litter source on the proportion of understory dominant combusted (Fig 6B). Litter sourced from triennially burned sites combusted differently in prescribed burns in annually and triennially burned environments: more triennial litter combusted in triennially burned sites than in annually burned sites (df = 13, t = 3.68, p = 0.063; Fig 6B). Similarly, in annually burned destination environments, a greater mass of annually-burned litter combusted than triennially-burned litter (df = 13, t = 4.11, p = 0.033; Fig 6B).

## Discussion

In this study, we found complex controls on decomposition that differed among landscape positions and among litter sources. Decomposition rates were consistently faster in the ecotones than in the uplands, suggesting an important role of soil moisture in decomposition in this ecosystem. Although destination environment influenced decomposition rates only inconsistently, when it did, burned sites had consistently high decomposition rates than fire-suppression sites.

### Effect of local environmental conditions on decomposition rates

Variation in fine-scale soil conditions has been proposed to explain differences in local decomposition rates [7, 42], but we found mixed evidence to extend this hypothesis into fire-dominated systems. We found significant differences in decomposition rates across landscape positions (i.e. upland versus ecotone), and thus overall evidence that environmental conditions influence decomposition. However, of the soil characteristics we measured (soil moisture, soil temperature, pH, soil nutrient availability) there were relatively few that differed among fire regimes (destination environments) within a landscape position. Perhaps as a consequence, the fire regime treatment did not have a consistent effect on decomposition rates. In the uplands, although suppression sites had slightly more acidic soil than burned sites, overall variation in soil physicochemical properties across burn regimes was minimal. This was surprising given the role of biochar in rapidly influencing soil physicochemical properties and nutrient availability [29, 31]. Given the relatively dry conditions of the upland soils, we expected that soil moisture would be an important regulatory factor for decomposition at this landscape position, and infrequent fires allow for the growth of shrubby vegetation, the buildup of forest floor litter, and increases in soil moisture retention. As such, we expected that variation in soil moisture across fire regimes would influence decomposition, and that decomposition rates would be higher in sites with fire-suppression. However, we found that patterns in soil moisture across fire regimes did not appear to correspond to litter decomposition, and in fact, decomposition was frequently higher in burned than suppression sites. However, soil moisture is highly variable throughout the year, and our soil moisture measurements were limited to relatively few measurements during the prior year’s growing season.

In contrast to the aggregated litter mixture, decomposition of the understory dominant litter differed across destination environments in uplands and ecotones. At both landscape positions, understory litter decomposed the fastest in triennially-burned sites. However, we could not explain these patterns with our measured environmental characteristics. Black carbon, which we did not measure, has been shown to influence microbial composition and activity via its effects on soil physicochemical properties [29, 30], although it’s impact on litter breakdown may be more limited [31]. Because we did not analyze the community composition of decomposing microbes [43], we cannot exclude the possibility that differences in decomposer abundance and/or composition between destination fire regimes influenced decomposition. We also cannot discount the possibility that differences in microbial activity influenced decomposition, although previous work has found that net mineralization and net nitrification rates were not influenced by time since fire [27]. While we observed fast decomposition of understory species in triennially burned destination environments, and fast decomposition of triennial understory litter, we did not detect significant interactions between litter source and destination environment showing enhanced decomposition of triennial litter decomposing in triennially burned sites, or “home” litter [44–46].

Overall, our results suggest that spatial variability in soil conditions independent of fire management history was the primary driver of decomposition rates in this landscape. Rather than across fire regimes, we found greater variability in soil characteristics across landscape positions, suggesting that a microtopographic assessment of a landscape must be accounted for in future studies when estimating decomposition rates. These differences may have resulted in significant differences in decomposition rates between upland and ecotone landscape positions. However, we might expect that sites managed for longer time under contrasting fire regimes would show an enhanced overall effect of fire regime on plant community composition, soil characteristics, and decomposition. That is, the duration of our contrasting fire regimes may have been too short to affect the soil conditions, such as soil moisture, which might have influenced decomposition rates.

### Effect of litter source on decomposition

A fire-driven shift in plant community composition, and consequently litter traits, was a secondary driver of decomposition rates in this system. Despite other studies identifying litter trait diversity as the strongest driver of litter carbon and nitrogen loss [47], we found a variable impact of litter source on decomposition. Leaf N content and, to a lesser extent, leaf C:N varied among litter sourced from different fire regimes, but we found inconsistent effects of litter source on decomposition. Although leaf C and N are commonly used to assess litter quality, litter source may have had a stronger role in determining litter decomposition if other measures of leaf decomposability, such as lignin content, were more strongly affected by fire history. In the uplands, triennially-burned *P*. *palustris* litter decomposed faster than annually-burned or fire suppressed *P*. *palustris* litter, despite no statistical difference in leaf C:N between triennially-burned and fire-suppressed *P*. *palustris* litter. This suggests that traits other than leaf N content may be more important determinants of decomposition in this system or for this species. Since *P*. *palustris* has serotinous cones and requires fire for successful reproduction, the response of this species to altered fire regimes may differ from that of fire-tolerant or non-pyrophytic species. Additionally, there was substantial variability in decomposition of *P*. *palustris* litter across sites. Although litter source was the sole significant predictor of *P*. *palustris* decomposition rates, the overall lack of consistent results suggests that for this species, trait variability associated with fire regime is not the sole predictor of decomposition.

In the ecotones, litter source influenced decomposition of the understory dominant species: triennially-burned litter (*A*. *tecta*) decomposed faster than annually-burned (*I*. *glabra*) or fire-suppressed (*G*. *frondosa*) litter. *A*. *tecta* also had higher leaf N content than *G*. *frondosa* and *I*. *glabra*. This suggests that fire-induced shifts in community composition towards N-rich species in triennially burned sites might enhance decomposition, but we cannot rule out the possibility that species identity, rather than the fire regime itself, is responsible for these patterns. However, in this system community composition is strongly shaped by fire frequency, so community composition, community trait values, and fire frequency are strongly linked. This finding supports extending the regulatory role of litter stoichiometry in decomposition models to disturbed systems [6]. However, *G*. *frondosa* decomposed slower than *I*. *glabra* despite having higher %N and lower C:N, emphasizing the continued need for improved decomposition frameworks that account for fine-scale nuances [5].

### Species specific differences in decomposition

*P*. *palustris*, the dominant overstory and titular species of this ecosystem, decomposed slower than all understory species in both uplands and ecotones. *A*. *stricta* was a dominant understory species in both suppression and annually-burned communities, but because it exhibited highly plastic litter quality traits (leaf C:N and %N), we expected variable decomposition rates. However, we found no difference in decomposition between the understory dominants in the uplands, nor was litter source a significant predictor of understory decomposition in the uplands. This could be explained by additional chemical traits related to litter quality that we did not measure, such as leaf cellulose and lignin content, that contribute to decomposition rates [48]. If these traits are implastic, the inclusion of *A*. *stricta* in multiple litter mixtures may have confounded our ability to detect a relationship between litter quality and decomposition. Alternatively, decomposition in the uplands may be so constrained by water availability, that an effect of litter substrate quality is only detectable under sufficient water availability. In this instance, interactions between litter quality and precipitation may result in highly temporally heterogeneous decomposition dynamics, particularly given the relatively high cellulose and hemicellulose content of *A*. *stricta* and *P*. *palustris* [48].

### Effects of fire characteristics on litter combustion

In frequently-burned systems, combustion is important for preventing litter accumulation and maintaining plant diversity [49]. We found that fires in triennially-burned sites combusted a greater proportion of *P*. *palustris* litter than fires in annually-burned sites, likely due to greater fire intensities in triennially-burned sites caused by greater fuel accumulation in unburned years [34]. We also found that understory species combusted differently in the same burn. In prescribed burns in annually-burned destination environments, litter sourced from annual and triennial sites combusted differently. This suggests that both upland and ecotone plant communities of annually-burned sites are more combustible than triennially-burned communities. In addition, the same triennially-burned litter combusted differently in annually burned and triennially burned destination environments. These results complement the well-accepted finding that plant species differ in their flammability [50], and that interspecific and intraspecific variation in flammability traits can also feedback to influence fire behavior [51]. This suggests that disturbance frequency can influence plant traits, which in turn can feed back to influence combustion dynamics, and consequently patterns of nutrient release in this ecosystem. In our highly simplified two-species litter mixtures, it appears that combustibility traits may have positive feedbacks on fire intensities: understory species of frequently-burned sites combust readily, hindering the accumulation of litter that would result in future high-intensity fires; communities in infrequently-burned sites do not combust readily, allowing for the accumulation of fuel to support a future high-intensity fire. Although previous work has emphasized growth rates and fire avoidance strategies as plant adaptations in fire-controlled systems [52, 53], our work suggests additional adaptation to fire through plastic leaf chemistry traits. Since fire dynamics are strongly linked to carbon and nutrient stocks across forest-savanna boundaries [54], an improved understanding of how plants traits mediate the effects of fire may have important implications for explaining the distribution of forest and savanna biomes [55, 56].

## Conclusions

In this study, we examined how decomposition of various litter sources differed across sites managed with different fire regimes. Overall, we found that topographic differences in soil characteristics and fire intensity exerted the strongest controls on litter loss and early-stage decomposition. Litter and soil characteristics, filtered through disturbance regime, exerted only secondary controls on decomposition. We found consistent interspecific differences in decomposition, highlighting the role of the disturbance regime in shaping the plant community more so than trait plasticity. Clearly, vegetation community composition and traits are influenced by both disturbances and soil conditions, and as such our results offer general support for the idea that interactions between micro-environmental conditions and litter carbon and nitrogen drive early-stage decomposition rates in a fire-maintained system. Further research is needed for a mechanistic understanding of these interactions, and to tease apart fire versus species effects on decomposition rates. In particular, an understanding of which plant traits influence decomposition and under which conditions may help us to understand how shifts in community composition may impact broad patterns of nutrient availability within an ecosystem. changes in carbon cycling in response to a shift in community composition. An understanding of the factors regulating decomposition in the context of disturbance is critical to developing accurate estimates of carbon storage and fluxes within and across ecosystems, particularly as climate change influences feedbacks between local community assemblages and environmental conditions.

## Supporting information

S1 Fig Mass remaining estimated from unburned versus full dataset. Data points are the proportion of mass remaining for suppression sites when estimated from curves fit to the full 12 months of data (x axis) vs the unburned 6 months of data (y axis). Dotted line is 1:1. R^2^ = 0.79, p<0.001).
(DOCX)

S1 Table Decomposition rates of plant litter. Decay rates (k), t, and p-values of the decomposition of P. palustris, the understory dominant and the aggregate litter mixture across treatments. Values were estimated by fitting exponential decay curves to unburned time points.
(PDF)
