# Supplementary material for: Effects of fire frequency on litter decomposition as mediated by changes to litter chemistry and soil environmental conditions
Source: PLoS One. 2017 Oct 12;12(10):e0186292. doi: 10.1371/journal.pone.0186292 (PMC5638519; doi:10.1371/journal.pone.0186292)
Supplement: S1 Fig — Data points are the proportion of mass remaining for suppression sites when estimated from curves fit to the full 12 months of data (x axis) vs the unburned 6 months of data (y axis). Dotted line is 1:1. R2 = 0.79, p<0.001). (DOCX) [file pone.0186292.s001.docx]

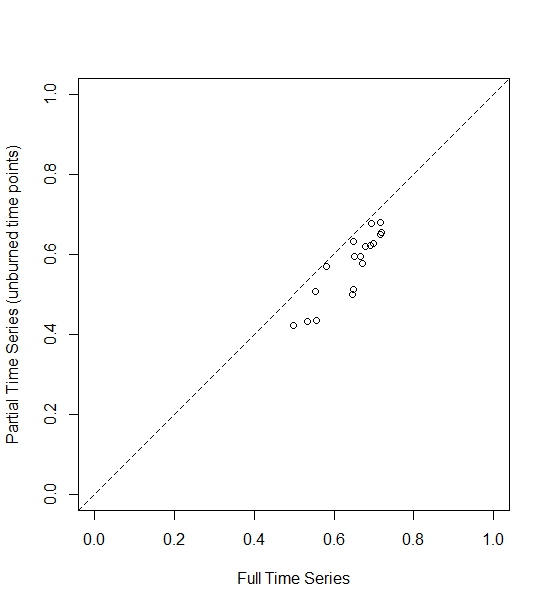
**Figure S1.** Data points are the proportion of mass remaining for suppression sites when estimated from curves fit to the full 12 months of data (x axis) vs the unburned 6 months of data (y axis). Dotted line is 1:1. R^2^=0.79, p<0.001).
