# Supplementary material for: Effects of fire frequency on litter decomposition as mediated by changes to litter chemistry and soil environmental conditions
Source: PLoS One. 2017 Oct 12;12(10):e0186292. doi: 10.1371/journal.pone.0186292 (PMC5638519; doi:10.1371/journal.pone.0186292)
Supplement: S1 Table — Decay rates (k), t, and p-values of the decomposition of P. palustris, the understory dominant and the aggregate litter mixture across treatments. Values were estimated by fitting exponential decay curves to unburned time points. (PDF) [file pone.0186292.s002.pdf]

Table S1. Decay rates (k), t, and p-values of the decomposition of P. palustris, the understory dominant and the aggregate litter mixture across treatments. Values were estimated by fitting exponential decay curves to unburned time points.

				Pinus palustris litter			Understory dominant litter			Aggregated litter		
Site	Landscape Position	Destination Environmen	Litter Source	*k*	t-value	p-value	*k*	t-value	p-value	*k*	t-value	p-value
3	ecotone	Tri	Ann	0.0400	5.0817	0.0739	0.0840	5.5670	0.0386	0.0616	5.7166	0.0472
3	ecotone	Tri	Tri	0.0440	5.4753	0.0364	0.1044	6.2353	0.0281	0.0701	6.2266	0.0309
3	ecotone	Tri	Supp	0.0285	3.8461	0.0959	0.0538	4.1669	0.0324	0.0407	4.2161	0.0532
3	upland	Tri	Ann	0.0339	4.4466	0.0325	0.0457	3.6930	0.0297	0.0405	4.1988	0.0274
3	upland	Tri	Tri	0.0396	5.0401	0.0200	0.0630	4.6511	0.0005	0.0498	4.9143	0.0098
3	upland	Tri	Supp	0.0378	4.8626	0.1512	0.0452	3.6631	0.0020	0.0414	4.2739	0.0356
5	ecotone	Ann	Ann	0.0419	5.2720	0.0062	0.0783	5.3400	0.0278	0.0573	5.4390	0.0221
5	ecotone	Ann	Tri	0.0450	5.5659	0.0447	0.1068	6.3008	0.0459	0.0756	6.5262	0.0428
5	ecotone	Ann	Supp	0.0405	5.1320	0.0274	0.0555	4.2632	0.0269	0.0483	4.8093	0.0306
5	upland	Ann	Ann	0.0242	3.3470	0.0031	0.0513	4.0280	0.0536	0.0339	3.6381	0.0325
5	upland	Ann	Tri	0.0314	4.1803	0.0312	0.0464	3.7366	0.0176	0.0405	4.1988	0.0274
5	upland	Ann	Supp	0.0227	3.1657	0.0069	0.0500	3.9538	0.0249	0.0346	3.7000	0.0169
6	ecotone	Supp	Ann	0.0237	7.2206	0.0032	0.0529	8.0222	0.0073	0.0365	8.0625	0.0050
6	ecotone	Supp	Tri	0.0364	9.8308	0.0006	0.0735	9.2432	0.0029	0.0523	9.9529	0.0013
6	ecotone	Supp	Supp	0.0287	8.3415	0.0015	0.0403	6.8769	0.0020	0.0339	7.6761	0.0027
6	upland	Supp	Ann	0.0280	8.1915	0.0004	0.0420	7.0548	0.0007	0.0361	8.0016	0.0003
6	upland	Supp	Tri	0.0258	7.7239	0.0025	0.0334	6.0885	0.0006	0.0300	7.0395	0.0008
6	upland	Supp	Supp	0.0222	6.8681	0.0049	0.0379	6.6119	0.0012	0.0277	6.6592	0.0025
7	ecotone	Ann	Ann	0.0323	4.2753	0.0417	0.0682	4.9031	0.0359	0.0493	4.8820	0.0379
7	ecotone	Ann	Tri	0.0276	3.7492	0.0813	0.0875	5.6942	0.0176	0.0555	5.3205	0.0269
7	ecotone	Ann	Supp	0.0249	3.4299	0.0001	0.0252	2.2670	0.0389	0.0259	2.8968	0.0177
7	upland	Ann	Ann	0.0242	3.3470	0.0031	0.0386	3.2362	0.1234	0.0316	3.4343	0.0651
7	upland	Ann	Tri	0.0261	3.5698	0.0995	0.0306	2.6782	0.0216	0.0299	3.2779	0.0409
7	upland	Ann	Supp	0.0228	3.1786	0.0377	0.0493	3.9118	0.0379	0.0348	3.7151	0.0424
17	ecotone	Supp	Ann	0.0243	7.3804	0.0021	0.0486	7.6687	0.0070	0.0362	8.0085	0.0047
17	ecotone	Supp	Tri	0.0258	7.7207	0.0018	0.0830	9.5999	0.0022	0.0491	9.6230	0.0015
17	ecotone	Supp	Supp	0.0241	7.3170	0.0013	0.0409	6.9422	0.0050	0.0333	7.5800	0.0023
17	upland	Supp	Ann	0.0250	7.5335	0.0016	0.0473	7.5613	0.0005	0.0358	7.9523	0.0010

Table S1. Decay rates (k), t, and p-values of the decomposition of P. palustris, the understory dominant and the aggregate litter mixture across treatments. Values were estimated by fitting exponential decay curves to unburned time points.

				Pinus palustris litter			Understory dominant litter			Aggregated litter		
Site	Landscape Position	Destination Environmen	Litter Source	*k*	t-value	p-value	*k*	t-value	p-value	*k*	t-value	p-value
17	upland	Supp	Tri	0.0286	8.3288	0.0026	0.0368	6.4883	0.0015	0.0324	7.4427	0.0020
17	upland	Supp	Supp	0.0265	7.8614	0.0006	0.0354	6.3301	0.0008	0.0307	7.1590	0.0005
19	ecotone	Supp	Ann	0.0365	9.8549	0.0016	0.0639	8.7630	0.0013	0.0494	9.6631	0.0009
19	ecotone	Supp	Tri	0.0395	10.3659	0.0007	0.0812	9.5406	0.0037	0.0580	10.4753	0.0015
19	ecotone	Supp	Supp	0.0451	11.2188	0.0022	0.0450	7.3482	0.0005	0.0451	9.1823	0.0008
19	upland	Supp	Ann	0.0187	5.9915	0.0009	0.0430	7.1528	0.0001	0.0305	7.1342	0.0002
19	upland	Supp	Tri	0.0232	7.1030	0.0183	0.0321	5.9167	0.0002	0.0276	6.6328	0.0037
19	upland	Supp	Supp	0.0195	6.2043	0.0006	0.0373	6.5444	0.0010	0.0279	6.6879	0.0001
28	ecotone	Tri	Ann	0.0357	4.6348	0.0530	0.0706	5.0094	0.0481	0.0541	5.2216	0.0538
28	ecotone	Tri	Tri	0.0353	4.5970	0.0064	0.1143	6.4946	0.0257	0.0357	3.7932	0.2417
28	ecotone	Tri	Supp	0.0307	4.1020	0.0527	0.0637	4.6883	0.0152	0.0467	4.6824	0.0372
28	upland	Tri	Ann	0.0283	3.8303	0.0526	0.0741	5.1672	0.0677	0.0479	4.7754	0.0714
28	upland	Tri	Supp	0.0240	3.3200	0.0268	0.0519	4.0614	0.1083	0.0355	3.7765	0.0248
31	ecotone	Ann	Ann	0.0346	4.5222	0.0169	0.0627	4.6357	0.0541	0.0467	4.6824	0.0372
31	ecotone	Ann	Tri	0.0364	4.7093	0.0336	0.1175	6.5714	0.0942	0.0694	6.1855	0.0419
31	ecotone	Ann	Supp	0.0290	3.9106	0.0298	0.0479	3.8264	0.0714	0.0391	4.0817	0.0634
31	upland	Ann	Ann	0.0266	5.7111	0.0003	0.0398	5.0800	0.0162	0.0331	5.5500	0.0012
31	upland	Ann	Tri	0.0378	4.8559	0.0079	0.0630	4.6511	0.0005	0.0478	4.7703	0.0029
31	upland	Ann	Supp	0.0211	2.9583	0.0177	0.0440	3.5895	0.0364	0.0313	3.4063	0.0085
15b	ecotone	Tri	Ann	0.0314	4.1803	0.0312	0.0687	4.9229	0.0544	0.0476	4.7558	0.0452
15b	ecotone	Tri	Tri	0.0243	3.3606	0.0264	0.0993	6.0848	0.0235	0.0584	5.5087	0.0278
15b	ecotone	Tri	Supp	0.0307	4.1020	0.0527	0.0375	3.1615	0.0750	0.0341	3.6531	0.0644
15b	upland	Tri	Ann	0.0274	3.7186	0.0113	0.0749	5.1979	0.0022	0.0515	5.0372	0.0070
15b	upland	Tri	Tri	0.0259	3.5406	0.0177	0.0394	3.2908	0.0049	0.0322	3.4837	0.0152
15b	upland	Tri	Supp	0.0228	3.1786	0.0377	0.0348	2.9769	0.0424	0.0283	3.1338	0.0526
